# Hypoxemia in trauma patients receiving two different oxygen strategies: a TRAUMOX2 substudy

**DOI:** 10.1186/s13049-025-01360-z

**Published:** 2025-03-18

**Authors:** Oscar Rosenkrantz, Tobias Arleth, Andreas Creutzburg, Louise Breum Petersen, Josefine Baekgaard, Stine Zwisler, Søren Mikkelsen, Markus Klimek, Lars Simon Rasmussen, Jacob Steinmetz

**Affiliations:** 1 Department of Anaesthesia, Centre of Head and Orthopaedics, Copenhagen University Hospital, Rigshospitalet, Copenhagen, Denmark; 2 Department of Clinical Epidemiology, Aarhus University Hospital and Aarhus University, Aarhus, Denmark; 3 The Prehospital Research Unit, Department of Regional Health Research, University of Southern Denmark, Odense, Denmark; 4 Department of Anaesthesiology and Intensive Care, Odense University Hospital, Odense, Denmark; 5 Department of Anaesthesiology, Erasmus University Medical Centre Rotterdam, Rotterdam, The Netherlands; 6 Danish Ministry of Defence Personnel Agency, Copenhagen, Denmark; 7 Danish Air Ambulance, Aarhus, Denmark

**Keywords:** Trauma, Hypoxemia, Pulse oximetry, Oxygen therapy, TRAUMOX2, Denmark

## Abstract

**Background:**

The randomized controlled trial, TRAUMOX2, compared early restrictive vs. liberal oxygen strategies for trauma patients. The objective of this substudy was to quantify the occurrence and duration of hypoxemic episodes during the trial’s eight-hour intervention.

**Methods:**

This observational substudy analyzed a subset of patients at two trial sites in Denmark. Continuous pulse oximetry recorded arterial oxygen saturation (SpO_2_) during the intervention. The primary outcome was the proportion of patients who had episodes of hypoxemia with SpO_2_ < 90% for at least five minutes. Additionally, the study assessed differences in the occurrence and duration of hypoxemia between the restrictive and liberal oxygen groups.

**Results:**

This substudy included 82 patients. After secondary exclusion, 60 patients (median age, 49 years [interquartile range 33–61] and 75% male) were analyzed. Three out of 60 patients (5%) had at least one episode of SpO_2_ < 90% for at least five minutes (95% confidence interval 1–14%); Two patients in the restrictive oxygen group and one in the liberal oxygen group. Two episodes occurred during initial resuscitation, and one episode occurred in the intensive care unit following a procedure related to thoracic injuries.

**Conclusions:**

In this substudy of 60 patients from the TRAUMOX2 trial, hypoxemia (SpO_2_ < 90% for at least five minutes) was observed in 5% of patients, with no difference between the restrictive and liberal oxygen groups. These findings suggest that, among trauma patients not already requiring continuous monitoring, such episodes of hypoxemia are relatively rare early post-trauma.

## Background

Injuries account for over four million deaths annually and are one of the leading causes of preventable deaths globally [1]. The Advanced Trauma Life Support (ATLS) guidelines recommend supplemental oxygen to all severely injured patients to prevent hypoxemia [2]. While preventing severe hypoxemia is critical, excess oxygen may cause hyperoxaemia [3–6], which may increase the risk of pulmonary complications and mortality [7–10]. This increased risk may, in part, be driven by reduced cardiac output caused by the formation of reactive oxygen species, effects that might occur within minutes [11]. The optimal dosage and duration of supplemental oxygen for trauma patients has not yet been specified, and the evidence for a beneficial effect of liberal supplemental oxygen is sparse [12, 13].

To address this gap, the TRAUMOX2 trial was conducted to compare early restrictive oxygen and liberal oxygen strategies after trauma and help determine whether a restrictive approach could reduce mortality and major respiratory complications. The trial did not detect statistically significant differences in death and/or major respiratory complications within 30 days [14]. In that trial, oxygenation was monitored by documenting arterial oxygen saturation hourly using pulse oximetry (SpO_2_), with the device usually placed peripherally on a finger. More episodes of hypoxemia were observed in the restrictive oxygen group. But this difference was not statistically significant after adjusting for multiple testing. Further, intermittent recording may be less sensitive in detecting hypoxemia than continuous monitoring [15–17].

There is no established threshold for when hypoxemia is harmful. Thus, while the TRAUMOX2 trial reported no differences in primary clinical outcomes between restrictive and liberal oxygen strategies, this substudy was conducted to provide a more detailed understanding of hypoxemia occurrence and duration in trauma patients in this setting. These insights may provide valuable insights for the broader trauma population, where the literature is sparse. Since the main trial collected SpO₂ data hourly, continuous monitoring in this substudy enabled a more accurate estimation of hypoxemic episodes of the trial population.

This investigation aimed to identify the occurrence and duration of hypoxemic episodes during the eight-hour intervention period of the TRAUMOX2 trial through continuous monitoring of SpO_2_ using portable pulse oximeters. We hypothesized that episodes with SpO_2_ less than 90% for at least five consecutive minutes would be observed in less than 10% of patients. Secondarily, we aimed to explore whether the occurrence and duration of hypoxemia differed between patients exposed to a restrictive vs. a liberal oxygen strategy.

## Methods

### Study design and setting

This observational substudy was part of the TRAUMOX2 trial, an international, randomized controlled trial that included 1508 patients randomly assigned 1:1 to a restrictive oxygen strategy (lowest dosage of supplemental oxygen that ensured an SpO_2_ of 94%) vs. a liberal oxygen strategy (12–15 L/min or fraction of inspired oxygen of 0.6–1.0) during the first eight hours after trauma [18].

For pragmatic reasons, this substudy included patients admitted to major trauma centers at two Danish trial sites: Copenhagen University Hospital and Odense University Hospital. These sites were selected due to their high inclusion rates, the availability of equipment, and the additional staff required to support the substudy.

The Danish Research Ethics Committee (H-21018062) and the Danish Medicines Agency (EudraCT 2021-000556-19) approved the TRAUMOX2 trial, which was also registered in the Capital Region of Denmark data controller’s register (P-2021-476), and at ClinicalTrials.gov (NCT05146700). Additional approvals, not relevant to this substudy, were also obtained.

### Selection of participants

This substudy screened consecutive trauma patients from the TRAUMOX2 trial at the two trial sites: adult trauma patients (18 years or older) with blunt or penetrating trauma and an anticipated hospital stay of more than 24 h. The TRAUMOX2 trial excluded patients suspected of carbon monoxide intoxication or those with cardiac arrest prior to randomization. Post-randomization, patients diagnosed with no or only minor injuries at the secondary survey in the trauma resuscitation room were excluded. Enrollment in the substudy was dependent on the availability of specific research staff and equipment, and we expected to include the majority between 8:00 AM and 4:00 PM on weekdays. The eight-hour TRAUMOX2 intervention started at randomization, either in the prehospital setting or on trauma center admission, while continuous SpO_2_ recording always commenced in the trauma resuscitation room. Patients included during the prehospital phase were expected to have shorter continuous SpO_2_ monitoring time in this substudy. Patients with less than four hours of continuous SpO_2_ data were secondarily excluded from this substudy. Consent followed Denmark’s deferred consent procedure for research in acute situations.

Data on the characteristics of patients from the main trial who were not included in the substudy but were recruited at the two centers during the main trial period (December 7, 2021, to September 12, 2023) were collected to evaluate potential selection bias.

### Measurements

After including a patient, an investigator attached a pulse oximetry sensor to one of the patient’s fingers or toes. The investigators prioritized extremities without traumatic lesions. We used Nellcor™ Portable SpO_2_ Patient Monitoring Systems, PM10N (Medtronic, Mansfield, MA, USA). The device measures SpO_2_ using photoplethysmography. This study used MAX-A-I Nellcor™ SpO_2_ adhesive sensors for single-patient use in adults. During the intervention, the devices recorded SpO_2_, date and time, heart rate, and alarm activation status. These variables were collected every second for a maximum of eight hours. The devices triggered an alarm if the SpO_2_ dropped below 85% or the heart rate fell below 40 bpm or exceeded 130 bpm. Any intervention resulting from such an alarm was at the discretion of the responsible clinician. The study group did not systematically collect information about interventions based on the alarms but conducted manual medical record reviews of the first hour after each alarm activation. Following the eight-hour intervention, an investigator removed the pulse oximeter and transferred data to a computer, pseudonymized it, and stored it on a secure drive.

Before analyzing the data, the authors removed artefacts from SpO_2_ measurement based on a method described by Haahr-Raunkjaer et al., which defined artefacts as changes in SpO_2_ greater than 4% per second [19]. Hypoxemic episodes where SpO_2_ dropped below specific thresholds were identified. Each successive reading below the limit increased the duration of these episodes by one second. Once SpO_2_ rose above the threshold again, the episode was considered to have ended.

### Outcomes

The primary outcome was the proportion of patients experiencing at least one episode with SpO_2_ less than 90% for at least five consecutive minutes. This threshold was chosen based on internal discussion in the author group, to capture sustained desaturation while minimizing the risk of artifacts, focusing on clinically relevant events. No clear threshold exists in the literature.

Secondary outcomes comprised the proportion of patients with one or more episodes with SpO_2_ less than 90% for at least two minutes, and the proportion with one or more episodes with SpO_2_ less than 85% for at least five minutes. The two-minute threshold was included as a sensitivity analysis to assess whether a notably higher number of patients would meet the hypoxemia criteria.

The study also assessed the cumulative time spent with SpO_2_ less than 90%, the duration of each episode with SpO_2_ less than 90%, and changes in oxygen treatment after an SpO_2_ alarm warning (SpO_2_ less than 85%).

### Analysis

Normally distributed variables were reported as means with standard deviations, and non-normally distributed variables as medians with interquartile ranges. Categorical data were presented as numbers with percentages and a 95% confidence interval where relevant. Confidence intervals for the absolute difference in proportions were calculated by the modified Wald method. To compare normally distributed data, the Student’s t-test was used. When comparing non-normally distributed data, the Mann-Whitney U test, with a Hodges-Lehmann estimator, was used as a nonparametric procedure. For proportion comparisons, Fisher’s exact test. All outcomes were compared between the two oxygen groups.

This substudy was designed to estimate the occurrence of clinically relevant hypoxemic episodes, defined as SpO_2_ less than 90% for at least five minutes, in trauma patients included in the TRAUMOX2 trial. If one patient out of 60 fulfilled this criterion, the risk of hypoxemia, including a 95% confidence interval, would be less than 10% using the modified Wald method. Data were collected until we had continuous monitoring for at least four uninterrupted hours on 60 patients. For the analyses, R statistical software (v4.3.1; R Core Team - Foundation for Statistical Computing, Vienna, Austria) was used.

## Results

### Characteristics of study participants

**Fig. 1:**
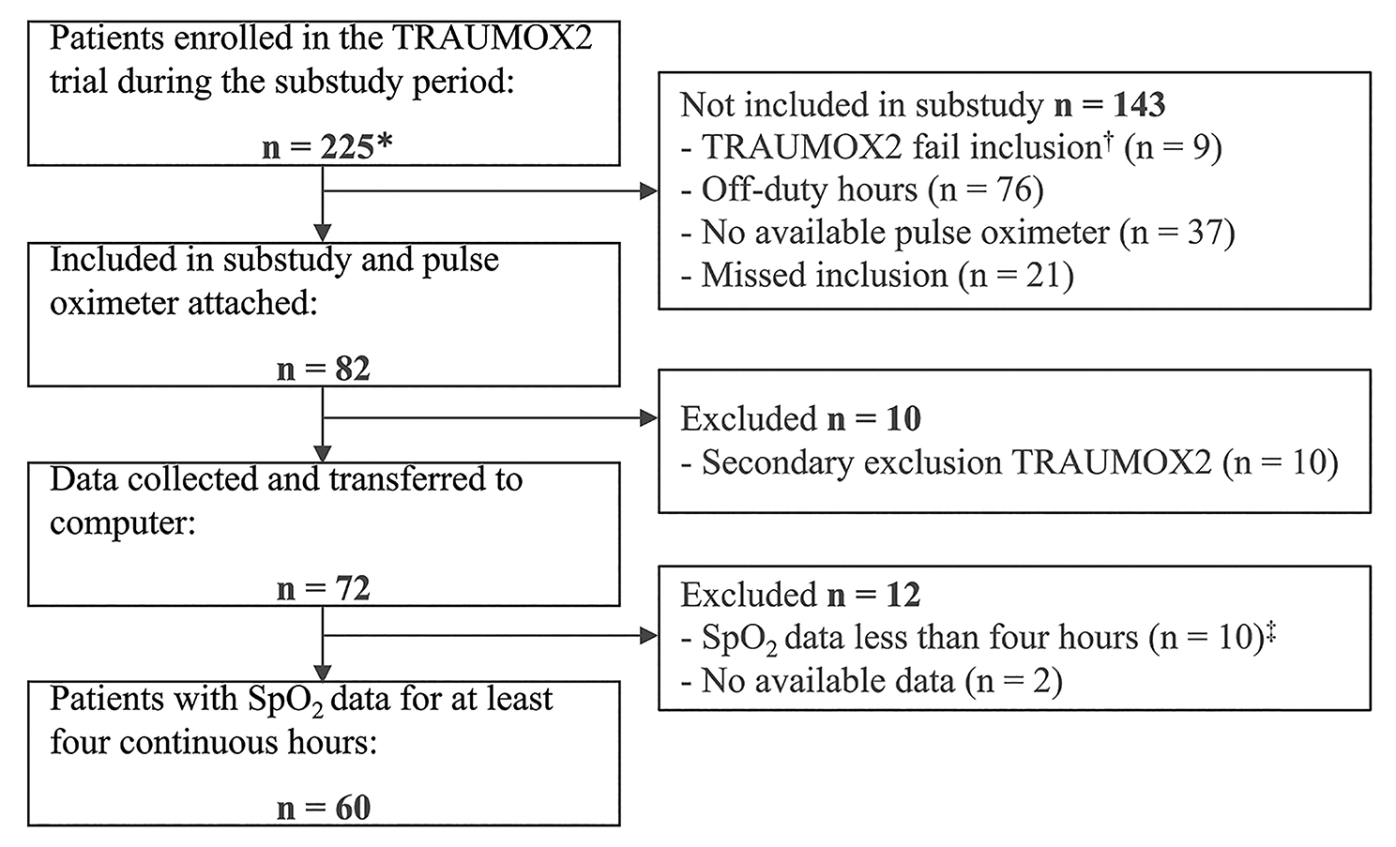
Flowchart of inclusion and data collection for trauma patients in this substudy Abbreviations: SpO_2_, arterial oxygen saturation measured by pulse oximetry *Odense University Hospital included patients in the substudy from June 20, 2022, forward †Patient included in TRAUMOX2 by mistake (randomized despite an exclusion criterion being met) ‡Seven times, the pulse oximeter was removed prematurely by patients or clinical health care staff, while the pulse oximeter ran out of battery three times.

Between March 28, 2022, and August 7, 2022, the trial sites activated 491 trauma teams, leading to the 225 TRAUMOX2 patients screened for this substudy. Pulse oximeter data collection began for 82 of these patients. After excluding 22 patients, 60 remained eligible for analysis (see Fig. 1 for details). The median age was 49 years (interquartile range 33–61), 75% were male, and median Injury Severity Score was 17 (interquartile range 9–24). In this sample of TRAUMOX2 patients, the liberal oxygen group included slightly older individuals and had a higher proportion of males, current smokers, patients with cardiovascular comorbidity, and patients with penetrating trauma. Other baseline characteristics were similar between the two groups. Throughout the main trial, 972 patients were included at the two trial sites but not in this substudy. Baseline characteristics were relatively similar overall, with values for the larger trial cohort generally falling between the two groups in the substudy, as shown in Table 1.

**Table 1:** Baseline characteristics of trauma patients allocated to either a restrictive or a liberal oxygen strategy

	Restrictive oxygen group (*N* = 30)	Liberal oxygen group (*N* = 30)	TRAUMOX2 trial cohort^∗^ (*N* = 972)
Age, median (IQR), years	46 (32–57) [*n* = 30]	56 (34–67) [*n* = 30]	49 (30–65) [*n* = 972]
Sex, No. (%), male	19/30 (63%)	26/30 (87%)	698/972 (72%)
Current smoker, No. (%)	4/26 (15%)	10/24 (42%)	255/739 (35%)
Lung disease comorbidity, No. (%)	2/30 (7%)	2/30 (7%)	87/972 (9%)
Cardiovascular comorbidity, No. (%)	4/30 (13%)	6/30 (20%)	191/972 (20%)
Body Mass Index, median (IQR), kg/m^2^	27 (22–28) [*n* = 26]	24 (22–28) [*n* = 25]	25 (22–28) [*n* = 797]
Predominant type of injury, No. (%)			
Blunt	28/30 (93%)	25/30 (83%)	849/972 (87%)
Penetrating	2/30 (7%)	5/30 (17%)	123/972 (13%)
Injury Severity Score, median (IQR)	17 (9–24) [*n* = 30]	17 (9–26) [*n* = 30]	14 (9–22) [*n* = 972]
First measured vital signs, No. (%)			
Systolic blood pressure < 90 mmHg	1/25 (4%)	2/28 (7%)	67/892 (8%)
Heart rate > 110 beats/min	3/26 (12%)	3/27 (11%)	135/894 (15%)
Respiratory rate > 24 breaths/min	3/20 (15%)	9/25 (36%)	122/700 (17%)
SpO_2_ < 90%	3/27 (11%)	8/30 (27%)	131/892 (15%)
Type of supplemental oxygen^†^, No. (%)			
No supplemental oxygen	18/30 (60%)	4/30 (13%)	396/972 (41%)
Nasal cannula	10/30 (33%)	2/30 (7%)	242/972 (25%)
Non-rebreather mask	1/30 (3%)	20/30 (67%)	375/972 (39%)
Intubated (during intervention)	14/30 (47%)	13/30 (43%)	447/972 (46%)

Abbreviations: IQR, interquartile range; SpO_2_, arterial oxygen saturation measured by pulse oximetry

^∗^Patients in the TRAUMOX2 trial at the two centers involved in this substudy, excluding the 60 patients included in this substudy

^†^Supplemental oxygen was recorded once every hour during the eight-hour intervention. Patients could receive different type of oxygen appliance. Therefore, count does not add to 100%

### Main results

Three patients (5%; 95% confidence interval 1–14%) experienced at least one episode of SpO_2_ less than 90% for at least five minutes. One patient in the liberal oxygen group had SpO_2_ less than 90% for 31 min with a nadir SpO_2_ of 76%. This patient, a traffic accident victim, sustained multiple injuries, including extensive thoracic and abdominal trauma, with an Injury Severity Score above 60. The patient arrived intubated at the trauma center, and the hypoxemic episode occurred while undergoing massive blood component transfusions during trauma resuscitation in the context of hemorrhagic shock, indicated by low blood pressure and a high lactate level. The other two patients with prolonged hypoxemic episodes were allocated to the restrictive oxygen group while hypoxemic. The first patient suffered extensive trauma to lower body extremities and head due to high-impact blunt trauma, with an Injury Severity Score above 40, and arrived intubated at the trauma center. This patient experienced two episodes during acute surgery, initially for 27 min with a nadir SpO_2_ of 71% and subsequently for 32 min with a nadir SpO_2_ of 88%. These episodes were managed under general anesthesia, with adjustments to FiO_2_ and ventilation settings in response to intraoperative conditions. The second patient in the restrictive group was also a traffic accident victim, with an Injury Severity Score above 20. The patient had one episode of six min with a nadir SpO₂ of 88% in the intensive care unit following a procedure related to thoracic injuries. This episode occurred shortly after intravenous opioid administration, which was characterized by a rapid but temporary respiratory compromise, as reflected by an increase in pCO_2_. Subsequent arterial blood gas analysis showed normal PaO_2_ and stable hemoglobin levels.

In total, six patients (10%; 95% confidence interval 4–20%) experienced at least one episode of SpO_2_ less than 90% for at least two minutes, while two patients (3%; 95% confidence interval 0–12%) experienced at least one episode of SpO_2_ less than 85% for at least five minutes.

Of the whole substudy population, the median cumulative time spent with SpO_2_ less than 90% was 24 s (interquartile range 0–146), while the median duration of each episode with SpO_2_ less than 90% was 10 s (interquartile range 5–17). The median continuous monitoring time was 6.9 h, equal to 86% of the eight-hour intervention period.

The SpO_2_ alarm (SpO_2_ less than 85%) was triggered 79 times in 21 patients. Manual reviews of the SpO_2_ curves surrounding the alarms identified the vast majority as artifacts rather than true hypoxemic events. The medical records showed no documentation of changes in oxygen treatment during the first hour after SpO_2_ alarms.

**Fig. 2:**
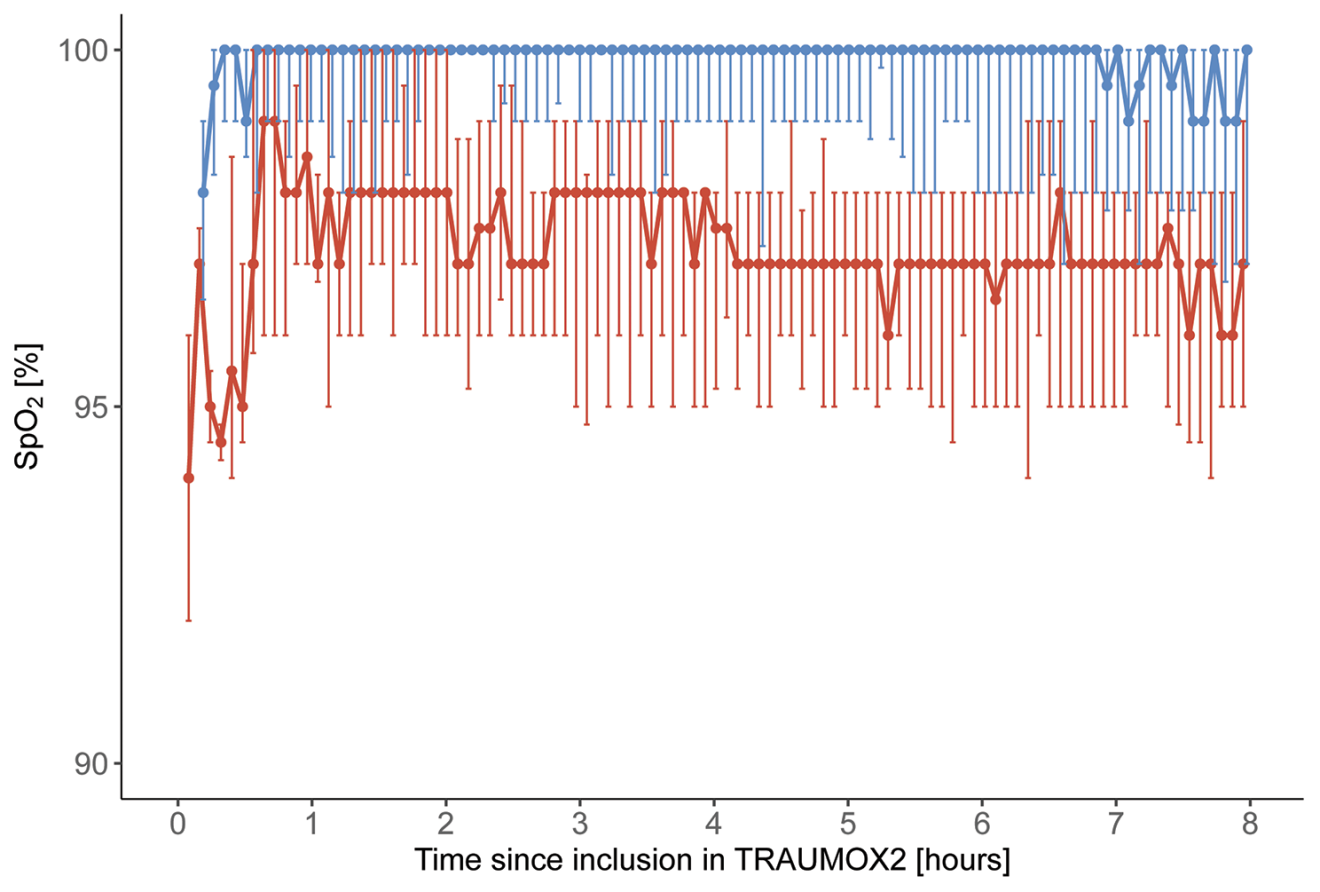
Pulse oximetry during the first eight hours after trauma Abbreviations SpO_2_, arterial oxygen saturation measured by pulse oximetry The horizontal lines show the median SpO_2_ with vertical bars representing the interquartile range (25th–75th percentiles) every five min

The SpO_2_ values were generally above 90% but showed a clear separation between the two groups (see Fig. 2).

A slightly longer duration of episodes with SpO_2_ less than 90% was observed in the liberal oxygen group with a median difference of 2 s per episode (95% confidence interval 1–4; *p* = 0.008). No other differences were found between the two oxygen strategy groups (see Table 2).

**Table 2:** Pulse oximetry for trauma patients allocated to either a restrictive or a liberal oxygen strategy

	Restrictive oxygen group (*N* = 30)	Liberal oxygen group (*N* = 30)	Difference (95% CI)	*P* value
SpO_2_ data, median (IQR), hours	6.8 (6.3–7.3)	7.1 (6.3–7.4)		
SpO_2_ < 90% for > 5 min	2 (7%)	1 (3%)	3% (-10–16%)	1.00^‡^
SpO_2_ < 90% for > 2 min	3 (10%)	3 (10%)	0% (-16–16%)	1.00^‡^
SpO_2_ <85% for > 5 min	1 (3%)	1 (3%)	0% (-12–12%)	1.00^‡^
SpO₂ <90% for any duration	25 (83%)	19 (63%)	19% (-3–40%)	
SpO₂ <85% for any duration	18 (60%)	15 (50%)	9% (-15–34%)	
SpO_2_ < 90% cumulative time, sec^∗^	58 (6–152)	13 (0–139)	12 (-4–57)	0.16^§^
SpO_2_ < 90% duration episodes, sec^†^	9 (4–15)	10 (5–22)	-2 (-4–-1)	0.008^§^

Abbreviations: CI, confidence interval; IQR, interquartile range; sec, seconds; SpO_2_, arterial oxygen saturation measured by pulse oximetry

Notes: For categorical outcomes, the 95% confidence interval for difference in proportions was calculated by the modified Wald method. For continuous outcomes, the median difference was computed as the Hodges-Lehmann estimate

^∗^The median of cumulative time per patient

^†^The median duration of all episodes per group

^‡^Fisher’s exact test

^§^Mann-Whitney U test

## Discussion

This substudy on hypoxemia among 60 TRAUMOX2 patients found that 5% (95% confidence interval 1–14%) of patients experienced at least one episode of hypoxemia with SpO_2_ less than 90% for at least five minutes. These findings do not rule out the possibility that the true occurrence of hypoxemia in the TRAUMOX2 population was more than 10%, as indicated by the higher boundary of the 95% confidence interval. Of the three patients with hypoxemia with SpO_2_ less than 90% for at least five minutes, two desaturated during the initial resuscitation in the trauma resuscitation room and one in the intensive care unit. Thus, none of the sixty patients experienced hypoxemia unattended in the hospital ward. Furthermore, there was no statistically significant difference in the number of hypoxemic episodes. However, we found a minor difference in the duration of episodes of SpO_2_ less than 90% between the two groups, with, on average, two-second longer episodes of hypoxemia in the liberal oxygen group, which likely has very limited clinical importance.

Pulse oximetry data exhibit pronounced variability. Also, SpO_2_ differs from arterial oxygen saturation assessed by arterial blood gas sampling (SaO_2_), and SpO_2_ accuracy decreases substantially for values below 90% [20]. Thus, the defined threshold of SpO_2_ less than 90% might not be optimal to detect actual SaO_2_ from just below 90%. The sensitivity analysis using a two-minute threshold for hypoxemia did not substantially alter the findings, as the proportion of patients experiencing hypoxemia remained similar across thresholds.

Several studies have used continuous pulse oximetry in combination with other variables to predict outcomes in trauma patients [21–24]. However, these studies do not report the duration of hypoxemic episodes, and we have not identified any studies reporting comparable data when using continuous pulse oximetry in trauma patients. Most studies on desaturation using continuous pulse oximetry primarily focus on the intraoperative or postoperative phase [25]. In 2010, Ehrenfeld et al. assessed intraoperative SpO_2_ in 95,407 adult non-cardiac surgical patients. They found that hypoxemia (SpO_2_ less than 90%) for at least two and five minutes occurred in 6.8% and 0.42% of their population, respectively [26]. In comparison, 10% and 5% of our patients had hypoxemic episodes with a duration of at least two and five minutes. However, Ehrenfeld et al. only collected data during surgery, with a mean duration of surgery of less than two hours. Thus, these patients were in a more controlled environment and monitored for a shorter time than our patients.

Postoperative hypoxemia in the ward after major abdominal surgery has been described by Duus et al. using continuous pulse oximetry [16]. They found that 58% of patients desaturated to SpO_2_ less than 92% for at least 60 consecutive minutes and that most hypoxemic episodes occurred on the second and third postoperative day. Thus, the first eight hours after trauma, which was the scope of our substudy, may not include the period where most hypoxemic episodes could be anticipated.

Some risk factors of postoperative oxygen desaturations might be opioid use and atelectasis [27, 28]. As atelectasis is one of the most common thoracic complications after blunt trauma [29] and opioid use is common, we might expect to see a similar pattern in trauma patients. However, this was beyond the scope of this paper.

In this substudy, most hypoxemic episodes occurred in the trauma resuscitation room and not in hospital wards. It is still unclear whether this type and duration of desaturations are associated with adverse patient outcomes and whether an altered oxygen treatment strategy would prevent such incidents. Oxygen desaturation may reflect underlying injuries or disease rather than an actual treatable condition. However, compared to studies on postoperative patients, only a few patients in the TRAUMOX2 population experienced desaturation using continuous SpO_2_ monitoring during the first eight hours after trauma. Thus, based on this sample of patients, the interventions of the TRAUMOX2 in a clinical setting seemed without a substantial risk of clinically relevant hypoxemic episodes. However, even with these reassuring findings, we cannot rule out that the true occurrence of clinically relevant episodes of hypoxemia in TRAUMOX2 patients is as much as 14%. Furthermore, our sample size was not calculated to detect differences between the two treatment groups. Combined with demographic differences such as older patients and more current smokers in the liberal oxygen group, we may have overlooked important differences in the occurrence of hypoxemia between the two groups, as type 2 error occurs more often in studies with small sample sizes and high data variability [30].

An important strength of this study is that we collected continuous SpO_2_ data for nearly seven hours (86%) of the eight-hour intervention period per patient, amounting to 1,451,500 measurements. We used small battery-powered pulse oximeters and adhesive single-use sensors to minimize the risk of missing data due to sensor removal and to limit the number of artifacts. To increase the data quality, only finger and toe sensors were used [31]. We used the same type of pulse oximeter on all patients in this study, and each device was calibrated before initial use. Further, we removed major artefacts before analysis.

### Limitations

This substudy has limitations. The study primarily enrolled patients during regular working hours. Including more patients at night might have revealed more episodes of hypoxemia, as previously observed in surgical patients [32]. This substudy only included a fraction (4%) of TRAUMOX2 patients and only from two trial sites, so it does not reflect the entire trial population. Information on oxygen titration in response to desaturations and treatment changes following alarms was sparsely documented in the medical records, limiting our ability to analyze these factors. The TRAUMOX2 population represents a selected group of trauma patients, as those with minor or no injuries were excluded. This selection of more severely injured patients may bias the results towards overestimating hypoxemic episodes compared to a broader trauma population. Conversely, the awareness of being in a trial might have influenced the behavior of patients and clinicians, increasing focus on oxygen therapy and desaturations, potentially underestimating the number of episodes and affecting the generalizability of the findings.

The study excluded 12 patients with less than four hours of continuous SpO_2_ monitoring. Most often (58%), the patient or clinical personnel removed the pulse oximeter prematurely, potentially introducing selection bias into the results. However, the direction of this potential bias is unclear. Additionally, patients in the liberal oxygen group of this substudy were more likely to be hypoxemic at baseline (27% vs. 11% in the restrictive group). This difference in baseline characteristics introduces a risk of bias in the results towards more frequent desaturations in the liberal oxygen group, which may have masked between-group differences. Lastly, by not muting the pulse oximetry alarm warning (SpO_2_ less than 85%) and thus alerting the staff, we might have influenced the treatment of patients who would otherwise, unnoticed, have experienced further oxygen desaturation. Even though no changes in oxygen treatment were found in the medical records following the alarm notifications, we might have recorded more episodes of severe hypoxemia if the alarm had been silenced. For example, Sun et al. reported 21% more desaturations with silenced alarms when using continuous pulse oximetry in non-cardiac surgery patients [17]. Lastly, this substudy was not powered to detect group differences. The sample size was based on estimating hypoxemic episodes in a sample of the overall trial population, which has limited the ability draw conclusions about between-group differences.

## Conclusion

This substudy of 60 patients from the TRAUMOX2 trial found that hypoxemia episodes defined as SpO_2_ less than 90% for at least five minutes occurred in 5% of patients during eight hours of continuous monitoring. There was no difference in the proportion having hypoxemic episodes between the restrictive and liberal oxygen groups. These findings suggest that, among trauma patients not already requiring continuous monitoring, such episodes of hypoxemia are relatively rare in the early post-trauma period.

## Data Availability

A deidentified dataset of substudy data will be made available upon reasonable request to the sponsor of the main trial and an approval for the use of data from the Danish Research Ethics committee after the long-term studies of six and 12-months follow-up of participants in the TRAUMOX2 trial have been published.

## Abbreviations

ATLS: Advanced Trauma Life Support
SaO_2_: arterial oxygen saturation measured by arterial blood gas sampling
SpO_2_: arterial oxygen saturation measured by pulse oximetry
